# Serially Quantifying *TERT* Rearrangement Breakpoints in ctDNA Enables Minimal Residual Disease Monitoring in Patients with Neuroblastoma

**DOI:** 10.1158/2767-9764.CRC-24-0569

**Published:** 2025-01-28

**Authors:** Jan F. Hollander, Annabell Szymansky, Jasmin Wünschel, Kathy Astrahantseff, Carolina Rosswog, Anne Thorwarth, Theresa M. Thole-Kliesch, Rocío Chamorro González, Patrick Hundsdörfer, Kathrin Hauptmann, Karin Schmelz, Dennis Gürgen, Julian M.M. Rogasch, Anton G. Henssen, Matthias Fischer, Johannes H. Schulte, Cornelia Eckert, Angelika Eggert, Marco Lodrini, Hedwig E. Deubzer

**Affiliations:** 1 Department of Pediatric Oncology and Hematology, Campus Virchow Klinikum, Charité – Universitätsmedizin Berlin, Berlin, Germany.; 2 Department of Experimental Pediatric Oncology, University Children’s Hospital of Cologne, Cologne, Germany.; 3 Center for Molecular Medicine Cologne, University of Cologne, Cologne, Germany.; 4 Experimental and Clinical Research Center (ECRC) of Charité, Max-Delbrück-Center of Molecular Medicine in the Helmholtz Association, Berlin, Germany.; 5 Max-Delbrück Center of Molecular Medicine in the Helmholtz Association, Berlin, Germany.; 6 Department of Pediatrics, Helios Klinikum Berlin-Buch, Berlin, Germany.; 7 Institute of Pathology, Campus Charité Mitte, Charité-Universitätsmedizin Berlin, Berlin, Germany.; 8 Charité - 3R, Charité – Universitätsmedizin Berlin, Berlin, Germany.; 9 Experimental Pharmacology and Oncology Berlin-Buch GmbH (EPO), Berlin, Germany.; 10 Department of Nuclear Medicine, Campus Virchow Klinikum, Charité – Universitätsmedizin Berlin, Berlin, Germany.; 11 Berlin Institute of Health (BIH) at Charité, Berlin, Germany.; 12 German Cancer Consortium (DKTK), Partner Site Berlin, and German Cancer Research Center (DKFZ), Heidelberg, Germany.; 13 Department of Pediatric Oncology and Hematology, University Hospital Tübingen, Tübingen, Germany.

## Abstract

Telomerase is reactivated by genomic *TERT* rearrangements in ∼30% of diagnosed high-risk neuroblastomas. Dismal patient prognosis results if the RAS/MAPK/ALK signaling transduction network also harbors mutations. We present a liquid biopsy–based monitoring strategy for this particularly vulnerable pediatric patient subgroup for which real-time molecular diagnostic tools are limited to date. Droplet digital PCR assays quantifying patient-individualized *TERT* rearrangement breakpoint copies, *ALK* copy numbers, and allelic *ALK* p.R1275Q mutation frequencies were applied to longitudinally collected liquid biopsies (peripheral blood and bone marrow [BM] plasma, *n* = 44 biosamples), the mononuclear cell fraction from BM and matched tumor samples. Marker detection was compared with current gold-standard diagnostics. Reanalysis of whole-genome and targeted panel sequencing data from 169 patients identified 64 *TERT*-rearranged neuroblastoma samples collected at initial and/or relapse diagnosis from 55 patients (254 total *TERT* rearrangement events). Detection and quantification of unique *TERT* rearrangement breakpoints in as little as 1 ng of total cell-free DNA in peripheral blood plasma improved therapy response assessment and early relapse detection in individual patients. Proof-of-concept is provided for minimal residual disease detection in the BM niche, from which relapses frequently arise, by analyzing unique *TERT* rearrangement breakpoints in BM plasma–derived cell-free DNA. *TERT* rearrangement breakpoints, as a single marker or combined with mutations in the RAS/MAPK/ALK signaling transduction network, can serve as robust and highly sensitive biomarkers for disease activity and spatially and temporally resolve disease better than current gold-standard diagnostics in individual patients with *TERT*-driven neuroblastoma.

**Significance:**

Real-time molecular monitoring of *TERT*-rearranged high-risk neuroblastoma is an unmet clinical need. We tested liquid biopsy-based assays for patient-individualized *TERT* breakpoint sequences to monitor disease in pediatric patients. Our digital PCR approach provides high resolution of spatial and temporal disease quantification in individual patients and is applicable for clinical routine.

## Introduction

Neuroblastoma, an embryonal tumor of neuroectodermal origin, is the most common extracranial solid malignancy of infancy and childhood. Approximately half of all pediatric patients newly diagnosed with neuroblastoma are at high risk of relapse despite aggressive multimodal therapy (1). Polychemotherapy usually provokes a good initial response; however, minimal residual disease (MRD) with dissemination of a few resistant tumor cells is at the root of relapses. Treating refractory or relapsed neuroblastoma remains challenging. The number of long-term survivors of high-risk disease has remained unsatisfactorily poor, with survival as low as 50% after first-line therapy and <20% after relapse.

A new mechanistic model of neuroblastoma yielded from whole-genome sequencing data showed that patients with poor prognoses had tumors harboring telomere maintenance mechanisms (2). Telomeres can be maintained via *MYCN* amplifications, *TERT* rearrangements, or alternative lengthening of telomeres. However, *TERT* rearrangements combined with mutations in the RAS/MAPK/ALK signal transduction network define a very high-risk neuroblastoma subgroup with a particularly poor patient outcome (2). Approximately 30% of newly diagnosed high-risk neuroblastomas have high *TERT* transcript levels and active telomerase caused by genomic *TERT* rearrangements (3, 4). Molecular in-time disease monitoring in this genetically defined high-risk subgroup is an urgent, as yet unmet, area of clinical need.

The invasive nature of surgical biopsies most often prevents their sequential application to monitor diseases. Single biopsies fail to reflect intratumor heterogeneity, cancer dynamics, and drug sensitivities that most likely change during disease evolution. Detection and characterization of cell-free nucleic acid molecules in biofluids can serve as minimally invasive liquid biopsy approaches. Liquid biopsies have a wide range of attractive clinical applications, including prognostication at diagnosis, treatment response assessment, MRD monitoring, early relapse detection, and targeted drug selection for personalized treatment. At least 35 studies, to date, report copy-number variations (CNV), single-nucleotide variants (SNV), indels, and methylation in cell-free ctDNA from infants and children with neuroblastoma (5). These studies showed that reappearance of *MYCN* and *ALK* CNVs in ctDNA preceded relapse diagnosis using established clinical parameters by up to 3 months in individual patients, supporting a future for ctDNA marker–based monitoring in routine aftercare programs (6). *ALK* p.F1174L and *ALK* p.R1275Q ctDNA marker surveillance also provided molecular resolution of spatial disease activity superior to tissue-based diagnostics, warranting validation in large clinical trials (6). Recently, two studies demonstrated that ctDNA sequencing can detect genomic evolution in neuroblastomas under first-, second-, or third-generation ALK inhibitor therapy (7, 8). The detected mutations created potential routes to bypass the ALK inhibitor, demonstrating the potential to identify clinically actionable collateral targets as ALK inhibitor resistance evolves (9).

In this study, we applied droplet digital PCR (ddPCR) protocols, applicable for the clinical routine, to monitor *TERT* rearrangement breakpoints in liquid biopsies as a single marker or combined with alterations in the RAS/MAPK/ALK signal transduction network. Our aim was to assess their potential for advanced molecular longitudinal disease monitoring in patients with very high-risk neuroblastoma.

## Materials and Methods

### Patient samples

Existing whole-genome (*n* = 20; refs. 2, 3) and targeted panel sequencing data (*n* = 169; ref. 10) from 169 tumor samples from patients with metastasized (stage M) high-risk neuroblastoma according to the International Neuroblastoma Risk Group (11) were reanalyzed to detect the number of neuroblastomas harboring *TERT* rearrangements. Matching peripheral blood and bone marrow (BM, Supplementary Table S1) samples were collected (local ethics approval: EA2/055/17) as previously described (6). Patients were treated at the Charité Berlin and registered in the NB2004-HR (NCT 00410631) clinical trial or the GPOH NB2016 Registry (DRKS00023442). Informed written patient/parent consent was obtained during trial/registry participation. White blood cells served as a source for germline DNA. Blood plasma was collected (local ethics approval: EA2/131/11) from 11 pediatric patients with nonmalignant conditions and a median age of 97.0 months (min-max: 28.7–246.0 months) as comparative controls. Peripheral blood was centrifuged at 1,900 × *g* for 7 minutes to separate plasma from cells (6). BM aspirates were centrifuged at 450 × *g* for 7 minutes to separate plasma from cells (6). All plasma samples were centrifuged a second time at 3,250 × *g* for 10 minutes to remove cellular debris before storage at −80°C (6). Mononuclear cells from BM aspirates were purified and quantified as previously described (12). In brief, BM samples were enriched for mononuclear cells by Ficoll density grade centrifugation, and 1 to 4 × 10^6^ mononuclear cell aliquots were stored at −20°C until further analysis.

### Assessing treatment response in patients

Overall response was defined as complete, partial, or minor response, and disease was assessed as stable or progressive according to the revised International Neuroblastoma Response Criteria (13). These criteria support an integrated response assessment for the primary tumor, soft tissues, bone metastases, and BM. Primary and metastatic tissue sites were assessed using [^123^I]*meta*-iodobenzylguanidine (mIBG) imaging or [^18^F]fluorodeoxyglucose PET/MRI and response evaluation criteria in solid tumors (RECIST) for mIBG-nonavid tumors. BM cytospins were analyzed in standard diagnostic using conventional cytology and anti-GD2 immunocytology (antibody 14G2A, BD Pharmingen, 5542272). Tumor marker assessments included in routine clinical practice were measuring blood levels of neuron-specific enolase and urine concentrations of the catecholamine metabolites, homovanillic acid, and vanillylmandelic acid. These clinical imaging and BM GD2 immunocytology methods are uniformly considered the gold standard for patient monitoring and to diagnose neuroblastoma relapse by The European Association for Neuroblastoma Research (SIOPEN) and the Children’s Oncology Group in North America.

### Animal experiments

Subcutaneous GI-ME-N cell (RRID: CVCL_1232) xenografts were created by injecting 5 × 10^6^ cells suspended in 100 µL Matrigel (Corning Life Sciences) into the flanks of NMRI-Foxn1nu/nu mice (RRID: IMSR RJ:NMRI-NUDE; *n* = 3). The subcutaneous patient-derived xenograft (PDX) model of patient P3 was generated in mice within the European Union–funded ITCC-P4 consortium (informed written patient consent is available from the consortium) and serially transplanted at least three times prior to implantation for PDX maintenance into the flanks of NOG mice (RRID: IMSR_TAC:HSCFTL-NOG, *n* = 5). Tumor size was measured daily using a caliper, and tumor volume was calculated using π/6 (width × height × depth). Mice were euthanized by cervical dislocation at day 30 or when tumor size exceeded 1,500 mm^3^. Total blood obtained through orbital puncture was collected in EDTA tubes and centrifuged at 1,900 × *g* for 7 minutes. Plasma was centrifuged at 3,250 × *g* for 10 minutes and pooled afterward. Plasma and tumors were stored at −80°C. Animal handling and care conformed to national and European Union regulatory standards, and experiments were approved by the local government agency (Landesamt für Gesundheit und Soziales Berlin).

### Cell culture

The human neuroblastoma cell lines, BE(2)-C (RRID: CVCL_0529), CLB-GA (RRID: CVCL_9529), GI-ME-N (RRID: CVCL_1232), IMR-32 (RRID: CVCL_0346), IMR-5 (RRID: CVCL_1306), Kelly (RRID: CVCL_2092), LAN-5 (RRID: CVCL_0389), LAN-6 (RRID: CVCL_1363), NB-1 (RRID: CVCL_GZ01), NBL-S (RRID: CVCL_2136), SH-EP (RRID: CVCL_0524), SH-SY5Y (RRID: CVCL_0019), SK-N-AS (RRID: CVCL_1700), SK-N-DZ (RRID: CVCL_1701), SK-N-FI (RRID: CVCL_1702), and TR14 (RRID CVCL_B474), were cultured in RPMI 1640 medium or DMEM (Thermo Fisher Scientific) supplemented with 10% FCS (GE Healthcare) and maintained at 37°C and 5% CO2 before harvest for subsequent DNA extraction. Conditioned medium was collected and centrifuged at 2,000 × *g* for 5 minutes, and the supernatant was stored at −80°C. Thawed conditioned medium was centrifuged at 2,000 × *g* for 5 minutes to clear debris, centrifuged at 20,000 × *g* for 5 minutes, and stored at −80°C until *TERT* rearrangement breakpoint or copy-number detection. Cell lines were authenticated by high-throughput SNP-based assays (14) and regularly monitored for *Acholeplasma laidlawii*, *Mycoplasma* species, and squirrel monkey retrovirus infections using high-throughput, multiplexed testing (15).

### Genomic and cell-free DNA preparation

Genomic DNA was extracted from tumor tissues using the QIAamp DNA Mini kit (Qiagen) or the Qiagen Puregene Core kit A (Qiagen), and quantified on a Thermo Fisher Fluorometer Qubit 2.0 (RRID: SCR_020553, Life Technologies, 6). Fragmentation was performed by adding 5U of AluI, HindIII or HaeIII restriction enzyme (New England Biolabs) to each ddPCR reaction (6). DNA was isolated from mononuclear cells (1–4 × 10^6^ cell aliquots) using the NucleoSpin Tissue kit (Macherey–Nagel). Total cell-free DNA (cfDNA) was purified using QIAamp Circulating Nucleic Acid kit (Qiagen) and then concentrated to 50 μL using DNA Clean and Concentrator-5 Kit (Zymo Research, 6). The cfDNA ScreenTape assay (Agilent) and the Agilent 4200 TapeStation System (RRID: SCR_018435) were used for quantification, and DNA fragments between 100 and 300 bp were considered to be total cfDNA (6).

### ddPCR

The Bio-Rad QX200 Droplet Digital PCR System (RRID: SCR_019707) was used to determine unique patient-specific *TERT* rearrangement breakpoints in uniplex reactions. *TERT* and *ALK* copy numbers and the *ALK* p.R1275Q (c.3824G>A) hotspot SNV with its corresponding wild-type sequence were analyzed in duplex and triplex ddPCR assays as previously described (6). False positive rates and limits of detection to reliably quantify tumor-specific CNVs (amplification, ≥8.01 gene copies; gain, 2.74–8.00 copies; normal diploid gene contingent, 1.50–2.73 copies) and the *ALK* p.R1275Q mutation were previously described (6). The following BioRad T100 Thermal Cycler (RRID: SCR_021921) programs were performed using optimized primer and probe concentrations for patient-specific *TERT* rearrangement breakpoints (Supplementary Table S2) and CNV/SNV detection and quantification (Supplementary Table S3): (i) GI-ME-N cell line–specific *TERT* breakpoint detection (denaturation: 95°C for 10 minutes; 60 cycles: 30 seconds at 94°C, 1 minute at 55°C, 2 minutes at 72°C; final denaturation: 10 minutes at 98°C); (ii) patient-specific *TERT* breakpoint detection (denaturation: 95°C for 10 minutes; 40 cycles: 30 seconds at 94°C, 1 minute at 58°C; final denaturation: 10 minutes at 98°C); (iii) CNV quantification (denaturation: 95°C for 10 minutes; 40 cycles: 30 sec at 94°C, 1 minute at 58°C; final denaturation: 10 minutes at 98°C); and (iv) *ALK* p.R1275Q SNV detection (denaturation, 95°C for 10 minutes; 40 cycles, 30 seconds at 94°C, 1 minute at 62.5°C; final denaturation, 10 minutes at 98°C). Breakpoints, CNVs, and mutant allele frequency were analyzed using QuantaSoft Analysis software, version 1.7.4.0917 (RRID: SCR_025696), and Bio-Rad QuantaSoft Analysis Pro, version 1.0.596 (RRID: SCR_025321). All ddPCR assays contained appropriate nontemplate, positive, and negative controls in each run. *TERT* rearrangement breakpoints in ctDNA-based liquid biopsies were determined by calculating the ratio of target molecule concentration A (copies/μL) times the purified sample volume (50 µL) to the total volume (mL) of liquid biopsy B ($TERT\;\text{rearrangement}\;\text{breakpoint}\;\text{copies}/\text{mL}\;=\;\frac{A\;\times \;50\;µl}{B}$). Genomic *TERT* rearrangement breakpoints were determined by calculating the ratio of total copy numbers in a reaction (copies/well), C, to the amount of input DNA (ng), D ($TERT\;\text{rearrangement breakpoint copies}/\text{ng}\;=\;\frac{C}{D}$). A sample was scored as positive for *TERT* breakpoint detection if the number of positive droplets was ≥4.

### Data availability

Targeted sequencing data from tumors have been deposited into the European Genome–Phenome Archive (EGA, https://ega-archive.org/; RRID: SCR_004944; Study ID EGAS00001007365; Dataset ID EGAD00001011088). Targeted sequencing data from cell lines are available at the NCBI Sequence Read Archive (http://www.ncbi.nlm.nih.gov/Traces/sra; RRID: SCR_004891; BioProject no. PRJNA979802; submission no. 13480648; SRR no. SRR24829337). Original ddPCR data generated in this study are available upon request from the corresponding author.

## Results

### ddPCR detects tumor- and patient-specific *TERT* rearrangements

We were interested whether the genomic breakpoints of *TERT* rearrangements have sequences that are unique to the neuroblastoma or metastases in a single patient, thus presenting precise target sequences capable of monitoring disease in serial liquid biopsies. We initially reanalyzed bulk sequencing data from a cohort of 169 neuroblastoma tumor samples (10) to detect the number of neuroblastomas harboring *TERT* rearrangements. Unique breakpoint sequences were identified in the *TERT* locus (chr5:1252600-1345000 encompassing the *TERT* and *CLPTM1L* genes; genome assembly GRCh37/hg19), the breakpoint region, according to Peifer and colleagues (3), in 64 neuroblastomas from 55 patients. Overall, 254 rearrangement events (508 breakpoints) had occurred in the tumor samples (Supplementary Fig. S1). Of these, 343 breakpoints were left as scars representing past mutation events in the *TERT* region (Supplementary Fig. S1A), 17 breakpoints were part of intrachromosomal rearrangements in chr.5 (Supplementary Fig. S1B), and 148 breakpoints indicated interchromosomal rearrangements involving chromosomes other than chr.5 (Supplementary Fig. S1C). Independently of their functional significance for disease in the patient, all 254 *TERT* rearrangement events detected could potentially be developed into patient-specific *TERT* breakpoint ddPCR assays to monitor the respective unique *TERT* rearrangement in patient biofluid samples.

**Figure 1:**
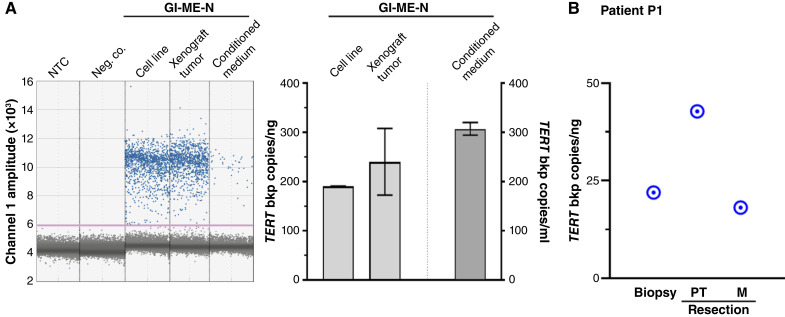
A ddPCR assay detecting unique *TERT* rearrangement breakpoints for improved in-time molecular disease monitoring. **A,** Representative original data output from the uniplex ddPCR assay designed to quantify copies of the *TERT* breakpoint unique to the GI-ME-N cell line (left). The 1D amplitude plot depicts event number (droplets) versus fluorescence amplitude with positive (blue) and negative (gray) droplets separated by the threshold line (pink). Quantification of *TERT* breakpoint copies detected in GI-ME-N cells grown *in vitro*, conditioned cell culture medium, and subcutaneous GI-ME-N xenografts grown in mice (mean ± SD, *n* ≥ 3). **B,** The *TERT* rearrangement breakpoint copies detected in genomic DNA from neuroblastoma tissue samples by ddPCR are shown for patient P1. Neg. co., negative control; NTC, nontemplate control; M, metastasis; PT, primary tumor.

To develop a ddPCR assay protocol and test its breakpoint detection and sensitivity for breakpoint copy quantification, we turned to the GI-ME-N neuroblastoma cell line, in which a genomic *TERT* rearrangement was previously reported (3) but not characterized on the level of the DNA sequence. Break-apart FISH (Supplementary Fig. S2A) confirmed the *TERT* breakpoint, which we identified both by whole-genome sequencing and panel sequencing (Supplementary Fig. S2B; Supplementary Table S4). Our GI-ME-N *TERT* breakpoint-specific uniplexed ddPCR assay clearly separated positive and negative droplets (Fig. 1A) and detected approximately 190 *TERT* breakpoint copies per ng in genomic DNA from cultured GI-ME-N cells, which was similar to copy numbers (240 ± 68) determined from the genomic DNA isolated from GI-ME-N cells, grown as subcutaneous xenografts in mice. cfDNA purified from a medium conditioned by GI-ME-N cells yielded 307 ± 13 *TERT* rearrangement breakpoint copies per mL, demonstrating that cfDNA is released by GI-ME-N cells in sufficient quantity to calculate breakpoint copy number from the fluid environment (Fig. 1A). In the background of human peripheral blood plasma, 1 ng GI-ME-N genomic DNA was required to reliably quantify the specific *TERT* rearrangement breakpoint (Supplementary Fig. S3A). This limit of detection sensitivity was determined by spiking 0.01 to 2.0 ng sonicated GI-ME-N DNA into blood plasma from pediatric patients with nonmalignant conditions. The 1 ng DNA spike-in was also sufficient to quantify *TERT* gene copy number, demonstrating that GI-ME-N cells belong to the rare cases in the panel of 16 neuroblastoma cell lines analyzed that harbored a *TERT* copy number gain (Supplementary Fig. S4).

We next applied our developed ddPCR assay protocol to assess breakpoints specific to the *TERT*-rearranged high-risk neuroblastoma diagnosed in patient P1 by *TERT* break-apart FISH (Supplementary Fig. S2A) and hybrid capture–based panel sequencing (Supplementary Tables S1 and S4). The patient-specific *TERT* breakpoint ddPCR assay required at least 2 ng genomic tumor DNA (Supplementary Fig. S3B) and detected breakpoint copies (Fig. 1B) at initial biopsy (22.0 copies/ng DNA) and postchemotherapy surgical resection (42.8 copies/ng primary tumor DNA, 18.1 copies/ng local lymph node metastasis DNA), thus validating our experimental approach in patient biomaterial samples (Supplementary Table S5). The novel ddPCR assay protocol was developed to detect *TERT* breakpoints which correlated well with results obtained with the independent methodologies, hybrid capture–based panel sequencing, and *TERT* break-apart FISH while only requiring input DNA amounts in the low nanogram range to reliably detect and quantify tumor-specific breakpoint copy numbers.

### *TERT* breakpoint monitoring in ctDNA captures disease activity in real time

To assess the power of our ddPCR assay for longitudinal monitoring of *TERT* breakpoints in patient samples, we analyzed tumor, blood, and BM samples from three patients with *TERT*-rearranged high-risk neuroblastoma (P2, P3, and P4; Supplementary Tables S1 and S4). Patient-specific ddPCR breakpoint assay design was feasible for all three patients (Supplementary Fig. S2), as was the case for patient P1. The fluid components from blood and BM were assessed and required at least 0.05 to 0.1 ng ctDNA as the starting material (defined by spike-in experiments in plasma; Supplementary Fig. S3C–S3E). The corresponding mononuclear cell fraction of every BM sample was also analyzed to directly determine the *TERT* breakpoints in neuroblastoma cells metastasized to the BM niche.

**Figure 2:**
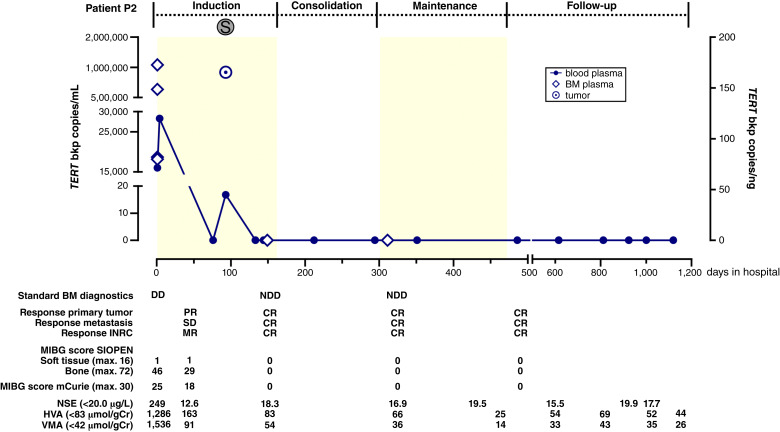
Clearance of *TERT* breakpoint sequence from ctDNA during induction therapy in patient P2. *TERT* rearrangement breakpoint copies (detected by ddPCR in purified ctDNA and genomic DNA) and selected clinical case information (below time line) are shown for longitudinally collected samples (type and time indicated in the graphical display) from patient P2. Light yellow and white backgrounds represent different treatment modules. BM, bone marrow; CR, complete remission; DD, detectable disease; HVA, homovanillic acid in urine/g creatinine; INRC, International Neuroblastoma Response Criteria; max., maximum; mIBG, [^123^I]*meta*-iodobenzylguanidine; MR, minor response; NDD, no detectable disease; NSE, neuron-specific enolase; PR, partial response; S, surgery; SD, stable disease; SIOPEN, The European Association for Neuroblastoma Research; VMA, vanillylmandelic acid in urine/g creatinine.

**Figure 3:**
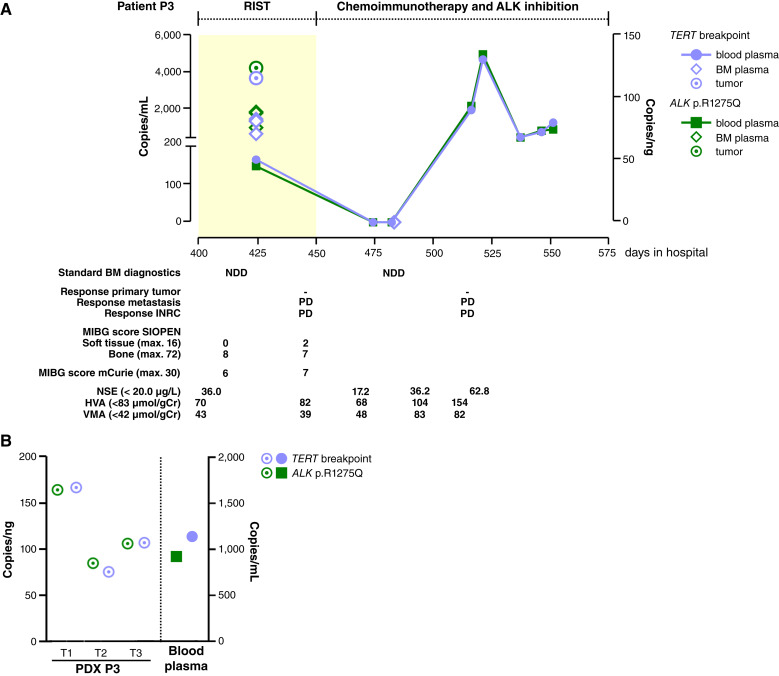
Targeted ctDNA-based *TERT* breakpoint and *ALK* p.R1275Q markers persist in patient P3. **A,** The graphical display integrates biosample and analysis type along the patient P3 course timeline for *TERT* rearrangement breakpoint (violet) and *ALK* p.R1275Q copies (green, top time line) and selected clinical case information (bottom time line). Light yellow and white backgrounds represent different treatment modules. **B, ***TERT* breakpoint and *ALK* p.R1275Q quantification in tumor tissue-derived genomic DNA and murine blood-derived ctDNA from the PDX model of P3. BM, bone marrow; HVA, homovanillic acid in urine/g creatinine; INRC, International Neuroblastoma Response Criteria; max., maximum; mIBG, [^123^I]*meta*-iodobenzylguanidine; NDD, no detectable disease; NSE, neuron-specific enolase; PD, progressive disease; PDX, patient-derived xenograft; RIST, molecularly targeted multimodal therapy consisting of metronomic courses of rapamycin/dasatinib and irinotecan/temozolomide; SIOPEN, The European Association for Neuroblastoma Research; T, tumor; VMA, vanillylmandelic acid in urine/g creatinine.

Longitudinal ddPCR-based assessment during the patient courses in two patients corresponded well to the current standard-of-care monitoring. Patient P2 responded well to the induction treatment and was in complete remission (CR) at the time of publication. Retrospective longitudinal *TERT* breakpoint monitoring in ctDNA from blood and BM plasma samples from patient P2 showed a molecular remission at the end of induction therapy (Fig. 2; Supplementary Table S6). *TERT* breakpoint concentration peaked at diagnosis in blood (28,315.9 copies/mL) and BM plasma (1,042,262.3 copies/mL), confirming ctDNA shedding into both compartments and demonstrating *TERT* ctDNA assay robustness for surveillance. The transient positive blood-based signal at day 92 (16.8 copies/mL) was likely attributable to primary tumor resection, which occurred on that day. Whether rapid ctDNA tumor marker clearance correlates with favorable event-free survival will require prospective validation studies in large patient cohorts. Rapid ctDNA marker clearance in patient P2 is contrasted by sustained *TERT* breakpoint marker persistence in patient P3 (Fig. 3A), reflecting the multimetastatic relapsed treatment-refractory disease in this patient. Molecular analysis of tumor tissue from patient P3 documented the *TERT* rearrangement breakpoint as well as the previously reported (6) *ALK* p.R1275Q mutation. Both markers were consistently detected in ctDNA from blood and BM plasma during second- and third-line treatment for progressive refractory disease (Fig. 3A; Supplementary Table S7). Longitudinally assessed *TERT* breakpoint and *ALK* p.R1275Q copy numbers were highly similar (Fig. 3A), possibly reflecting their origins in the same neuroblastoma clone. Both markers were also detected in tumor tissue and blood plasma from the PDX mouse model derived from tumor samples from patient P3 (Fig. 3B), confirming suitability of this model for preclinical liquid biopsy studies. Our findings demonstrate that *TERT* breakpoint ctDNA-based patient monitoring can reflect therapy success or failure, and that this real-time disease monitoring approach can be multiplexed to add further clinically important ddPCR markers to create more comprehensive monitoring.

### *TERT* breakpoint analysis in ctDNA detects neuroblastoma relapse

**Figure 4:**
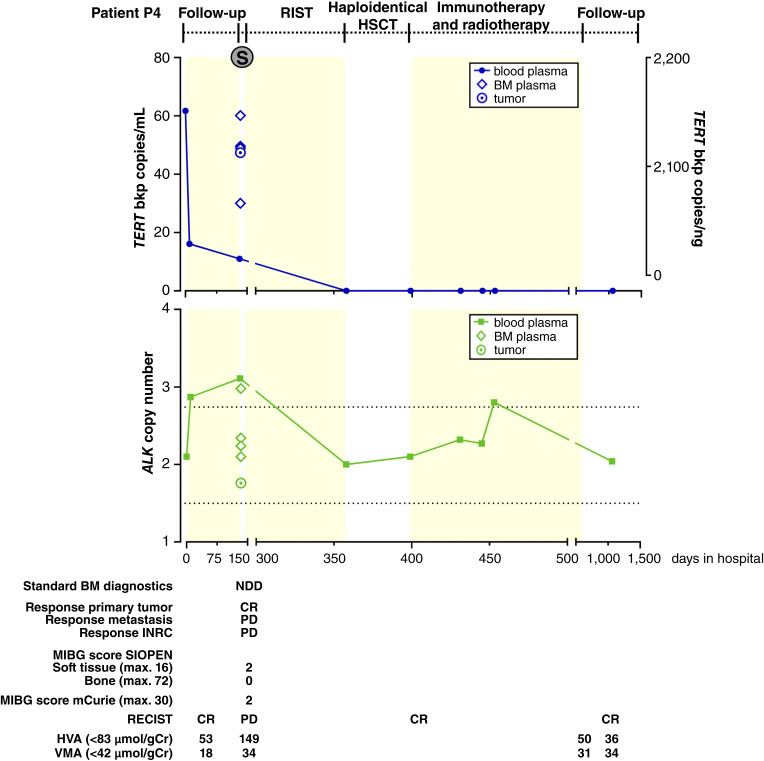
Longitudinal *TERT* breakpoint monitoring in ctDNA detects molecular relapse of high-risk neuroblastoma in patient P4. *TERT* breakpoint copies (top) and *ALK* copy numbers (bottom; both detected by ddPCR in purified ctDNA and genomic DNA) as well as selected clinical case information (below* time line*) are shown for longitudinally collected samples (type and time indicated in the graphical display) from patient P4. Light yellow and white backgrounds represent different treatment modules. BM, bone marrow; CR, complete remission; HSCT, hematopoietic stem cell transplantation; HVA, homovanillic acid in urine/g creatinine; INRC, International Neuroblastoma Response Criteria; max, maximum; mIBG, [^123^I]*meta*-iodobenzylguanidine; NDD, no detectable disease; PD, progressive disease; RECIST, response evaluation criteria in solid tumors; RIST, molecularly targeted multimodal therapy consisting of metronomic courses of rapamycin/dasatinib and irinotecan/temozolomide; S, surgery; SIOPEN, The European Association for Neuroblastoma Research; VMA, vanillylmandelic acid in urine/g creatinine.

Tumor tissue samples from initial diagnosis and first relapse in the fourth patient (patient P4) were previously analyzed by targeted sequencing, reporting a partial *ALK* gain (6). Reanalysis of the targeted sequencing data from patient P4 (Supplementary Table S4) detected a genomic *TERT* rearrangement in addition to the partial *ALK* gain that was confirmed by *TERT* break-apart FISH (Supplementary Fig. S2A). Both markers were retrospectively compared in ctDNA from longitudinal blood and BM plasma samples (Fig. 4). The first blood sample analyzed during routine follow-up was 15.7 months after the end of treatment. Plasma from this sample showed diploid *ALK* status, which correlated with the clinically assessed first CR in patient P4 [Fig. 4 (bottom); Supplementary Table S8; time point termed day 0 in hospital]. However, the *TERT* breakpoint ddPCR assay revealed a molecular relapse (61.7 copies/mL) at this time point that went undetected [Fig. 4 (top); Supplementary Table S8]. Blood plasma collected 10 days later showed both the *ALK* gain (2.87 total copies) and patient-specific *TERT* breakpoint (16.1 copies/mL; Fig. 4; Supplementary Table S8). Routine imaging performed on day 122 during routine follow-up documented a locoregional relapse in the primary tumor region (Fig. 4). No disease was detected on day 134 in BM diagnostics performed for restaging at the time of relapse (Fig. 4). The ctDNA analysis in BM plasma from the four different BM collection sites in the iliac crest documented diploid *ALK* copy numbers at three sites and an *ALK* gain at the fourth site (Fig. 4 (bottom); Supplementary Table S8). In contrast to this, the *TERT* breakpoint was detected in all four sites [30.0–60.1 copies/mL; Fig. 4 (top); Supplementary Table S8]. Patient P4 reached a second CR, and the cfDNA became negative both for the *ALK* gain and *TERT* breakpoint during relapse treatment (Fig. 4). Altogether, molecular monitoring of the *TERT* breakpoint in the two compartments (peripheral blood and BM) retrospectively detected the neuroblastoma relapse in patient P4 earlier and more accurately than (i) MRI and ^123^I-mIBG clinical imaging and BM GD2 immunocytology (considered the gold standard for neuroblastoma monitoring in Europe and North America) and (ii) the previously reported cfDNA-based *ALK* copy number assessment (6). While monitoring, the peripheral blood for *TERT* breakpoint copies in cfDNA detected the locoregional relapse earlier than routine follow-up diagnostics. Molecular assessment of the BM revealed a BM involvement that went completely undetected by routine measures and was only visible at one of the four different sampling sites using the *ALK* copy number ddPCR assay. The higher temporal and spatial resolutions of *TERT* breakpoint detection reported supports this assay’s potential in future management protocols for patients with very high-risk *TERT*-rearranged neuroblastomas.

### *TERT* breakpoint detection in BM plasma detects MRD in the BM niche

**Figure 5:**
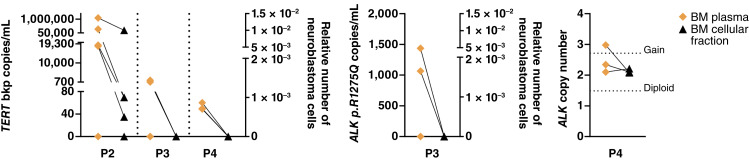
Bone marrow (BM) plasma is sufficient to detect *TERT* breakpoint, *ALK* p.R1275Q mutation, and *ALK* copy numbers in the novel ddPCR assay. Comparative analysis of *TERT* breakpoints, *ALK* p.R1275Q mutation, and *ALK* copy numbers in cell free DNA in the separated liquid component (BM plasma) and the cellular fraction samples from BM aspirates from patients P2 (days 0, 148, and 310 after diagnosis), P3 (days 423 and 482 after diagnosis), and P4 (day 134 in hospital). Pairs from the same patient sample are connected by lines.

As neuroblastoma relapses frequently arise in the BM (16, 17), monitoring clearance of all initially infiltrating neuroblastoma cells from the BM niche is essential information to adapt therapy intensity with the goal to eradicate MRD. We assessed the potential to use BM plasma to monitor disease, a patient biosample fraction that is currently most often discarded and not used in diagnostics. We reanalyzed *TERT* breakpoint copy detection by ddPCR in BM plasma–derived ctDNA and genomic DNA from the mononuclear cell fraction from the same BM aspirates from patients P2, P3, and P4 (Supplementary Tables S6–S8). Additional markers assessed by ddPCR such as *ALK* R.1275Q from patient P3 (Supplementary Table S7) and *ALK* copy number from patient P4 (Supplementary Table S8) were included in this analysis. Comparing marker detection in BM plasma versus BM mononuclear cells yielded a concordant result in 12 of 20 ddPCR analyses (Fig. 5). In 8 of 20 ddPCR analyses (40%), the respective marker was detected in the ctDNA from BM plasma but not the genomic DNA extracted from the matched BM mononuclear cell specimen (Fig. 5). Our data support proceedings to comparatively assess plasma and mononuclear cell fractions from BM aspirates prospectively and are blinded in an accessory investigation to a clinical trial (results not reported to the treating physicians) to test and refine the best monitoring combinations, including *TERT* breakpoint copy-number detection and additional molecular MRD markers, applicable for at least some patients in the trial cohort.

## Discussion

In this study, we demonstrate the feasibility of detecting patient-specific *TERT* rearrangement breakpoints in ctDNA from blood and BM plasma using ddPCR. Liquid biopsy–based detection of the patient-specific *TERT* rearrangement breakpoints mirror the presence of disease in individual patients, indicating the potential for this assay to monitor disease in patients with high-risk and very high-risk neuroblastomas harboring *TERT* rearrangements. *TERT* breakpoint ctDNA ddPCR is a novel patient-specific diagnostic tool complementary to routine investigative procedures. We demonstrate sensitive MRD detection in real time in our pilot study. Detection sensitivity was higher in patient biofluids than genomic DNA from tumor samples and the mononuclear cell fraction of BM aspirates.

Although the patient number presented here is low, it must be stressed that current standard-of-care monitoring using imaging detects relapse only after nests of cancer cells (>10^9^ generally regarded as the lower limit for imaging) are established. As yet, no molecular assays accurately detecting, and quantifying MRD exist for the neuroblastoma high-risk subgroup molecularly defined by the presence of *TERT* aberrations. This molecular disease subgroup includes patients at high-risk and very high–risk for relapse (2), emphasizing the urgent need for more sensitive detection particularly for this patient group. Here, we included data from patients with very high–risk disease based on their molecular profiles of genomic *TERT* rearrangements combined with mutations in the RAS/MAPK/ALK signaling transduction network. Our data are promising and, given the poor survival and current lack of existing in-time molecular disease monitoring tools for infants and children with *TERT*-rearranged neuroblastoma, support the blinded evaluation of our ddPCR-based assay in an accompanying prospective assessment to a clinical trial for neuroblastoma treatment.

The previously realized high sensitivity of ctDNA-based *MYCN* copy number and *ALK* mutation detection in respective patients with *MYCN*-amplified or ALK-driven disease (6–8, 18) has provided hope that new liquid biopsy–based approaches could better capture MRD and molecular relapses in patients with *TERT*-rearranged neuroblastoma. Using ctDNA in BM plasma would also complement MRD diagnostics using the cellular component from BM aspirates routinely collected during treatment and follow-up (19). To adequately compare detection power in patient biofluids, we developed and tested *TERT* breakpoint ddPCR on ctDNA in peripheral blood and BM plasma in parallel with genomic DNA from the cellular components in the BM aspirates and (multiregion) tumor biopsies collected at initial diagnosis or relapse from individual patients. Following proof of technical feasibility using ctDNA purified from medium conditioned by the GI-ME-N cell line, this study established uniplexed patient-specific ddPCR assays for four different *TERT* breakpoints and observed highly individual patient courses. The marker persisted in patient P3, with mixed therapy response and progressive disease, whereas ctDNA-based marker clearance from plasma correlated with CR and survival in patient P2. Reappearance of the *TERT* breakpoint in blood plasma preceded standard-of-care relapse diagnosed at 4.5 months in patient P4, supporting a future for ctDNA-based *TERT* breakpoint patient monitoring in routine aftercare programs. *TERT* breakpoints performed better than *ALK* copy numbers as a marker to detect early relapse in longitudinal ctDNA samples from these four patients, detecting the relapse 10 days earlier than *ALK* copy numbers (6). The tumor specificity of the *TERT* rearrangement breakpoint may make it a better marker than a gene amplification, because the *ALK* copy-number gain plays against the normal diploid background of cfDNA derived from nonmalignant cells. We conclude that multiplexing markers will provide optimal disease surveillance for patients treated for very high–risk neuroblastoma, as performed for patient P3 by combining *TERT* breakpoint and *ALK* p.R1275Q marker surveillance in ctDNA.

**Figure 6:**
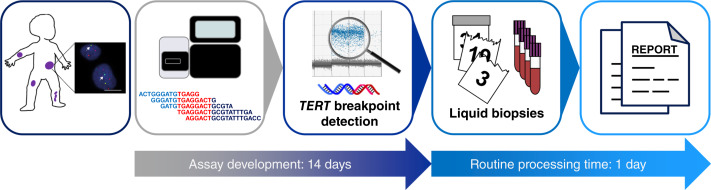
Schematic workflow to use patient-specific *TERT* rearrangement breakpoints as a clinical diagnostic marker. Panels show steps in the workflow from left to right: *TERT* break-apart FISH confirms *TERT*-rearranged disease, next-generation sequencing of biopsied neuroblastoma tissue supports ddPCR assay design, *TERT* breakpoint ddPCR analysis applied to longitudinally collected biosamples, samples are collected and processed, and report is generated. Routine processing time (after successful assay design) includes sample processing and laboratory report generation.

*TERT*-rearranged neuroblastoma cases are routinely identified by break-apart FISH (20). Targeted sequencing is necessary to characterize the exact *TERT* breakpoint sequence involved in the *TERT* rearrangement for patient-specific *TERT* breakpoint ddPCR assay design, which takes ∼15 working days (workflow schematically summarized in Fig. 6). Established assays are ready for use on liquid biopsies, or any other starting biomaterial, and can be processed in 1 day, demonstrating their feasibility for the clinical routine. Recently, *MYCN* breakpoint detection in ctDNA from patients with *MYCN*-amplified neuroblastomas was reported (21). Whether quantifying the *MYCN* amplicon breakpoint outperforms quantifying *MYCN* copy number in ctDNA, as now performed using a single standardized ddPCR assay (6) within the SIOPEN HR-NBL2 trial (NCT04221035) and the upcoming SIOPEN pragmatic clinical trial (MONALISA) to monitor neuroblastoma relapse with liquid biopsy–sensitive analysis for patients with MYCN-driven tumors, remains to be assessed in comparative studies. *TERT* rearrangements, however, have no other method of detection besides the patient-specific assay we report here. *TERT* rearrangements are usually not accompanied by *TERT* CNVs (2, 3), in contrast to high-level *MYCN* amplifications in aggressive neuroblastoma cell clones harboring *MYCN* breakpoints (22–24). The efforts associated with our approach are justified to enable sensitive detection of *TERT* rearrangements in this molecular high-risk subgroup of neuroblastoma, which does not overlap with cases harboring *MYCN* amplifications and includes both high-risk and very high–risk disease (2).

The concept of MRD monitoring was originally developed for children with acute lymphoblastic leukemia and has revolutionized their treatment, as therapy intensity has since been adjusted not only to the leukemic blast genetic profile at initial or relapse diagnosis but also to therapy response (25, 26). MRD monitoring in patients with acute lymphoblastic leukemia uses PCR assays applied to cells collected in BM aspirates. In this study, we show that *TERT* breakpoint copies and additional alterations such as the *ALK* p.R1275Q mutation and an *ALK* gain can be detected both in BM plasma–derived ctDNA and in genomic DNA extracted from the matched mononuclear cell fraction in BM. All three molecular markers were detected at higher frequency in BM plasma than in the mononuclear cell fractions from the same BM aspirate. Most importantly, sensitivities of our ddPCR approach were higher than the current diagnostic standard, GD2 immunocytology. The relative detection strength of liquid and cellular BM fractions needs confirmation in a large patient cohort and, combined with comparative testing of other developed ddPCR-based marker tests, could form a consensus about the most useful combinations for monitoring in the clinical routine and trials.

The *TERT* promoter is mutated at high frequencies in multiple cancers affecting adults (27–30). Studies have demonstrated the potential to detect and quantify *TERT* mutations in blood-based ctDNA from patients with melanoma (27) and brain tumors (28) and correlated detection of *TERT* promoter mutations with unfavorable response to first-line treatment regimens containing immunotherapy to treat hepatocellular carcinoma (29, 30). Likewise, *TERT* promoter methylation can be monitored in liquid biopsies from patients with urothelial carcinomas (31). Adapted to the principle of fusion gene breakpoint detection in pediatric Ewing sarcomas harboring *EWS-FLI1* fusions (32) and *KMT2A*- or *ETV6*/*RUNX1*-positive acute lymphoblastic leukemias (33, 34), we provide proof-of-concept for the similar detection of *TERT* rearrangement breakpoints in a highly aggressive and often deadly molecular high-risk neuroblastoma subgroup. This is a novel liquid biopsy–based in-time monitoring concept for infants and children with *TERT*-rearranged neuroblastoma with and without additional mutations in the RAS/MAPK/ALK signaling network. Its superior performance to the current clinical gold standard for monitoring in individual patients warrants its blinded inclusion in prospective investigations accompanying clinical trials for a rapid validation in large patient cohorts.

## Supplementary Material

Supplementary Data supplementals pdf file

Figure S1 Figure S1

Figure S2 Figure S2

Figure S3 Figure S3

Figure S4 Figure S4
