# Supplementary material for: Serially Quantifying TERT Rearrangement Breakpoints in ctDNA Enables Minimal Residual Disease Monitoring in Patients with Neuroblastoma
Source: Cancer Res Commun. 2025 Jan 28;5(1):167–77. doi: 10.1158/2767-9764.CRC-24-0569 (PMC11774142; doi:10.1158/2767-9764.CRC-24-0569)
Supplement: Supplementary Data — supplementals pdf file [file crc-24-0569_supplementary_data_suppsd.pdf]

SUPPLEMENTARY DATA FOR

Serial *TERT* rearrangement breakpoint quantification in circulating tumor DNA enables minimal residual disease monitoring in patients with neuroblastoma

Jan F. Hollander*, Annabell Szymansky*, Jasmin Wünschel, Kathy Astrahantseff, Carolina Rosswog, Rocío Chamorro González, Anne Thorwarth, Theresa M. Thole-Kliesch, Patrick Hundsdörfer, Kathrin Hauptmann, Karin Schmelz, Dennis Guergen, Julian M. M. Rogasch, Anton G. Henssen, Matthias Fischer, Johannes H. Schulte, Cornelia Eckert, Angelika Eggert†, Marco Lodrini†, Hedwig E. Deubzer†

*,† Authors contributed equally to this work.

Table of Contents

**Materials and Methods**	starting p. 2
**Tables**	starting p. 4
**References**	starting p. 12
**Figures**	starting p. 13

MATERIALS AND METHODS

Hybrid capture-based panel sequencing

Formalin-fixed, paraffin-embedded or fresh-frozen tumor material was histologically evaluated by an experienced pathologist, and regions with at least 10% tumor cell content were macrodissected from a total of 10 - 15 10 µM thick sections using the Leica LMD6 Laser Microdissection microscope system (Leica Microsystems, RRID:SCR_024658). DNA was automatically extracted using the Maxwell® 16 DNA Purification Instrument (Promega, RRID:SCR_020254) and the Maxwell® 16 FFPE Plus LEV DNA Purification Kit (Promega) and mechanically sheared by ultrasonic acoustic energy (Covaris ME220 Focused Ultrasonicator; RRID:SCR_019818). A total of 100 – 200 ng DNA were subjected to a custom hybrid capture-based next-generation sequencing panel assay for neuroblastoma (HpH Institute for Hematopathology Hamburg, Germany) to detect rearrangements/gene fusions, point mutations, small insertions deletions and copy number alterations in a single assay as previously described (1). In brief, target-specific hybrid capture biotinylated probes were designed for the genomic regions of interest using the custom SureSelectXT design (Agilent Technologies, RRID:SCR_013575). Selected baits ensured optimal coverage of all relevant genomic regions. After enrichment, targeted fragments were amplified (clonal amplification) and sequenced in parallel using Illumina's MiSeq System (RRID:SCR_016379) with an average mean sequencing depth of 1000-2500x (unfiltered reads, 2). Data were analyzed using a custom analysis pipeline that assessed quality of the raw sequencing reads and then aligned reads to the GRCh37 (hg19) human reference genome assembly (1). Discordant read pairs were subsequently extracted and the structural variants and their breakpoints were localized. Positions of all detected alterations were recorded with all supporting data evidence (spanning and encompassing sequencing reads).

Fluorescence in situ hybridization

TERT break-apart FISH was performed as previously described (3) using customized digoxigenin and biotin-labeled FISH probes and streptavidin-Alexa-555 conjugate (1:500 in CAS-block, Thermo Fisher Scientific Cat# s21381, RRID:AB_2307336) and anti-digoxigenin-FITC antibody (1:500 in CAS-block, Roche Cat# 11207741910, RRID:AB_514498). Slides were counterstained with VECTASHIELD HardSet Mounting Medium with DAPI (Vector Laboratories Cat# H-1200, RRID:AB_2336790). Microscopy was performed using a Leica DM5500 B Automated Upright Microscope System (RRID:SCR_018896) with Cytovision

(Leica, version 7.7, RRID:SCR_025697) and Fiji (version 1.52p, RRID:SCR_002285) software tools. Images were captured using a CV-M4+CL digital camera (JAI).

TABLES

Table S1. Clinical and molecular characteristics in patients with distant metastatic neuroblastoma[1]

Patient identifier	Age at diagnosis [days]	Biological Sex	EFS status[2]	OS status[3]	*TERT* status[4]	*MYCN* status[5]	1p36 status[6]	*ALK* status[7]
P1	1012	M	0	0	rearranged	U	U	*ALK* p.R1231W
P2	886	M	0	0	rearranged	U	U	U
P3	64	M	1	1	rearranged	U	U	*ALK* p.R1275Q
P4	1544	F	1	0	rearranged	U	U	gain

[1] Distant metastatic disease (stage M) according to the International Neuroblastoma Risk Group Staging System (4)
[2] 0 = no event; 1 = event
[3] 0 = alive; 1 = died from disease
[4] *TERT* gene status analyzed by hybrid capture-based panel sequencing (1)
[5] Genomic *MYCN* gene copies analyzed by fluorescence in situ hybridization, Southern blot and ddPCR (5)
[6] Chromosome 1p36 region analyzed for potential losses by fluorescence in situ hybridization and ddPCR
[7] *ALK* gene status analyzed by hybrid capture-based panel sequencing (1) and ddPCR (5)
EFS = event-free survival, OS = overall survival, M = male, F = female, U = unaltered

Table S2. Primers and probes for uniplex ddPCR protocols to detect source-specific *TERT* rearrangement breakpoints

Cell line/ Patient identifier	Primer/Probe	Sequence (5'-3')	Concentration [nM]
GI-M-EN	Tr-GI-M-EN-for	CGACTGCAACACTCCCCG	900
	Tr-GI-M-EN-rev	AGCGCTGCCTGAAACTCG	900
	Tr-GI-M-EN-probe	FAM-CTCAGCGACCCCTCCCCTCC-BHQ1	300
P1	Tr-P1-for	TGTTGTCTGCCTTGGGTTGT	900
	Tr-P1-rev	TTTGTCCAGCATCAGGGAGG	900
	Tr-P1-probe	FAM-TGCTGGCTTCTCTGACCAAAAGGT-BHQ1	250
P2	Tr-P2-for	TTCAGGGTCTGAAGTTGTGG	900
	Tr-P2-rev	CTTTCCATTGCCCCCACG	900
	Tr-P2-probe	FAM-AGCTTGTTCTCTCCCCCAACCCCACCT-BHQ1	250
P3	Tr-P3-for	GGAGCCTCCTCAATATTCTTCAAT	900
	Tr-P3-rev	CATTCCTTGGCTCATAGCTCC	900
	Tr-P3-probe	FAM-CAGGTGCAGATTCAAAGCGTGAG-BHQ1	250
P4	Tr-P4-for	CAAAGGTGCACACACACACG	900
	Tr-P4-rev	CTCGGACTGCAGAGCTAGGA	900
	Tr-P4-probe	FAM-ACGTGCACGCTGGAAGTCGG-BHQ1	250

ddPCR = droplet digital PCR

Table S3. Primers and probes for duplex or triplex ddPCR protocols to assess *ALK* and *TERT* copy number and *ALK* p.R1275Q mutant allele frequency

Primer/Probe	Sequence (5'-3')	Concentration [nM]
AFF3-for[1]	CACCTAGCATGTGTGGCATT	900
AFF3-rev[1]	GCAGATCCAGGTCGTTGAAG	900
AFF3-probe[1]	HEX-AACAACTCTTTCTGTCCCCCT-BHQ1	125
ALK -for[2]	CTTGTCCTCTGACTCTTCTCG	900
ALK -rev[2]	CAAGACTCCACGAATGAGC	900
ALK -probe[2]	FAM-TCACAGCTCCGAATGTCCTG-BHQ1	250
ALK^{1275}-for[3]	GTCCAGGCCCTGGAAGAG	900
ALK^{1275}-rev[3]	GGGGTGAGGCAGTCTTTACTC	900
ALK^{R1275Q} probe[3]	FAM-TTCGGGATGGCCCAAGACAT-BHQ1	250
ALK^{1275} probe[3]	HEX-TTCGGGATGGCCCGAGACAT-BHQ1	250
NAGK-for[4]	TGGGCAGACACATCGTAGCA	900
NAGK-rev[4]	CACCTTCACTCCCACCTCAAC	900
NAGK-probe[4]	HEX-TGTTGCCCGAGATTGACCCGGT-BHQ1	350
TERT-for	CGCAAGGCCTTCAAGAGC	900
TERT-rev	CCCAGAGACACACATCCTGG	900
TERT-probe	FAM-AGGTTCACGTGTGATAGTCGT-BHQ1	125

[1] Peitz *et al.*, 2020 (5)
[2] Lodrini *et al.*, 2022 (7)
[3] Adapted from Combaret *et al.*, 2015 (8)
[4] Adapted from Gotoh *et al.*, 2005 (9)
ddPCR = droplet digital PCR

Table S4. Genomic location of *TERT* rearrangement breakpoints

Cell line/ patient identifier[1]	Breakpoint rearrangement partner 1			Breakpoint rearrangement partner 2			Mean coverage of targeted NGS assay[2]
	Genomic position	Gene	Gene region	Genomic position	Gene	Gene region	
GI-ME-N	chr5:1,295,192	intergenic	Upstream *TERT*	chr19:58,607,052	*ZSCAN18*	Intron 1	1624x
P1	chr5:1,254,866	*TERT*	Intron 10	chr6:22,472,329	intergenic	Upstream *HDGFL1*	1023x
P2	chr5:1,321,572	*CLPTM1L*	Intron 12	chr10:8,145,607	intergenic	Downstream *GATA3*	1382x
P3	chr5:1,321,645	*CLPTM1L*	Intron 12	chr7:107,087,658	*COG5*	Intron 6	1420x
P4	chr4:8,792,662	intergenic	Downstream *HMX1*	chr5:1,296,135	intergenic	Upstream *TERT*	1816x

[1] Patient code
[2] Every target position is decoded by targeted NGS on average 1023-1816 times
Chr = chromosome, NGS = next-generation sequencing

Table S5. Overview of *TERT* breakpoint copies detected in neuroblastoma tissue samples from patient P1

Days in hospital	*TERT* breakpoint [copies/ng][1]	
	Primary tumor	Metastasis
3	22.0	
204	42.8	18.1

[1] Detected by droplet digital PCR

Table S6. Overview of *TERT* breakpoint copies detected in longitudinal biosamples from patient P2

Days in hospital	*TERT* breakpoint copies[1]			
	**Blood plasma** [copies/mL]	**BM[2] plasma** [copies/mL]	**BM[2] cellular fraction[3]**	**Tumor** [copies/ng]
0	16,048.9	635,204.9<br>18,689.5<br>18,176.0<br>1,042,262.3	0.001<br>0.001<br>0.0005<br>0.01	
3	28,315.9			
75	0			
92	16.8			165.6
132	0			
143	0			
148		0<br>0<br>0	0<br>0<br>0	
211	0			
293	0			
310		0	0	
350	0			
485	0			
615	0			
811	0			
923	0			
1000	0			
1119	0			

[1] Copies of the *TERT* breakpoint were detected by droplet digital PCR
[2] Bone marrow samples collected from different locations in the iliac crest
[3] The relative number of marker-positive neuroblastoma cells per 1 x 10^6 mononucleated cells
BM = bone marrow

Table S7. Overview of ddPCR-based *TERT* rearrangement breakpoint and *ALK* p.R1275Q mutation detection in longitudinally collected biosamples from patient P3

Days in hospital	*TERT* breakpoint copies				*ALK* p.R1275Q copies				
	Blood plasma [copies/mL]	BM[1] plasma [copies/mL]	BM[1] cellular fraction[2]	Tumor [copies/ng]	Blood plasma [copies/mL]	BM[1] plasma [copies/mL]	BM[1] cellular fraction[2]	Tumor [copies/ng]	Germline[3] [copies/ng]
423	170.3	1540.3<br>720.9	0<br>0	117.1	152.4 (7.6)[4]	1437.0 (5.6)[4]<br>1066.3 (6.7)[4]	0<br>0	125.5 (43.7)[4]	0 (0)[4]
473	0				0 (0)[4]				
481	0				0 (0)[4]				
482		0	0			0 (0)[4]	0		
515	2034.0				2250.0 (22.0)[4]				
520	4800.4				5081.5 (22.1)[4]				
536	544.7				522.9 (10.6)[4]				
545	826.6				900.0 (13.9)[4]				
550	1338.3				963.0 (14.6)[4]				

[1] Bone marrow samples collected from different locations in the iliac crest
[2] Shown is the relative number of marker-positive neuroblastoma cells per 1x 10^6 mononucleated cells.
[3] Assessed in DNA from peripheral blood (collected at diagnosis) cellular fraction
[4] Values in parentheses indicate mutant allele frequency, with 0% indicating no *ALK* p.R1275Q mutation was detected

BM = bone marrow, ddPCR = droplet digital PCR

Table S8. Overview of ddPCR-based *TERT* breakpoint and *ALK* copy number detection in longitudinal biosamples from patient P4

Days in hospital	*TERT* breakpoint copies				*ALK* copy number		
	**Blood plasma** [copies/mL]	**BM**[1] **plasma** [copies/mL]	**BM**[1] **cellular fraction**[2]	**Tumor**[3] [copies/ng]	**Blood plasma**	**BM**[1] **plasma**	**BM**[1] **cellular fraction**[2]
0	61.7				2.10		
10	16.1				2.87		
132	11.0				3.11		
134		49.6	0			2.34	2.10
		30.0	NA[4]			2.24	NA[4]
		60.1	0			2.98	2.08
		48.9	0			2.10	2.21
137				2112.7<br>582.5<br>354.5<br>345.2<br>584.3<br>17.0<br>437.6<br>549.0			
358	0				2.00		
445	0				2.27		
1062	0				2.04		

[1] Bone marrow samples collected from different locations in the iliac crest
[2] The relative number of marker-positive neuroblastoma cells per 1 x 10^6 mononucleated cells
[3] Multi-region tumor biopsies as previously described[6]
[4] No sample was available for analysis
BM = bone marrow, NA = not analyzed

REFERENCES USED IN SUPPLEMENTARY TEXT & TABLES

1. Szymansky A, Kruetzfeldt LM, Heukamp LC, Hertwig F, Theissen J, Deubzer HE, et al. Neuroblastoma risk assessment and treatment stratification with hybrid capture-based panel sequencing. J Pers Med 2021;11:691.
2. Muller JN, Falk M, Talwar J, Neemann N, Mariotti E, Bertrand M, et al. Concordance between Comprehensive Cancer Genome Profiling in Plasma and Tumor Specimens. J Thorac Oncol 2017;12:1503-11.
3. Peifer M, Hertwig F, Roels F, Dreidax D, Gartlgruber M, Menon R, et al. Telomerase activation by genomic rearrangements in high-risk neuroblastoma. Nature 2015;526:700-4.
4. Monclair T, Brodeur GM, Ambros PF, Brisse HJ, Cecchetto G, Holmes K, et al. The International Neuroblastoma Risk Group (INRG) staging system: an INRG Task Force report. J Clin Oncol 2009;27:298-303.
5. Peitz C, Sprussel A, Linke RB, Astrahantseff K, Grimaldi M, Schmelz K, et al. Multiplexed quantification of four neuroblastoma DNA targets in a single droplet digital PCR reaction. J Mol Diagn 2020;22:1309-23.
6. Schmelz K, Toedling J, Huska M, Cwikla MC, Kruetzfeldt LM, Proba J, et al. Spatial and temporal intratumour heterogeneity has potential consequences for single biopsy-based neuroblastoma treatment decisions. Nat Commun 2021;12:6804.
7. Lodrini M, Graef J, Thole-Kliesch TM, Astrahantseff K, Sprüssel A, Grimaldi M, et al. Targeted analysis of cell-free circulating tumor DNA is suitable for early relapse and actionable target detection in patients with neuroblastoma. Clin Cancer Res 2022;28:1809-20.
8. Combaret V, Iacono I, Bellini A, Brejon S, Bernard V, Marabelle A, et al. Detection of tumor ALK status in neuroblastoma patients using peripheral blood. Cancer Med 2015;4:540-50.
9. Gotoh T, Hosoi H, Iehara T, Kuwahara Y, Osone S, Tsuchiya K, et al. Prediction of MYCN amplification in neuroblastoma using serum DNA and real-time quantitative polymerase chain reaction. J Clin Oncol 2005;23: 5205-10.

FIGURES

Supplementary Figure S1

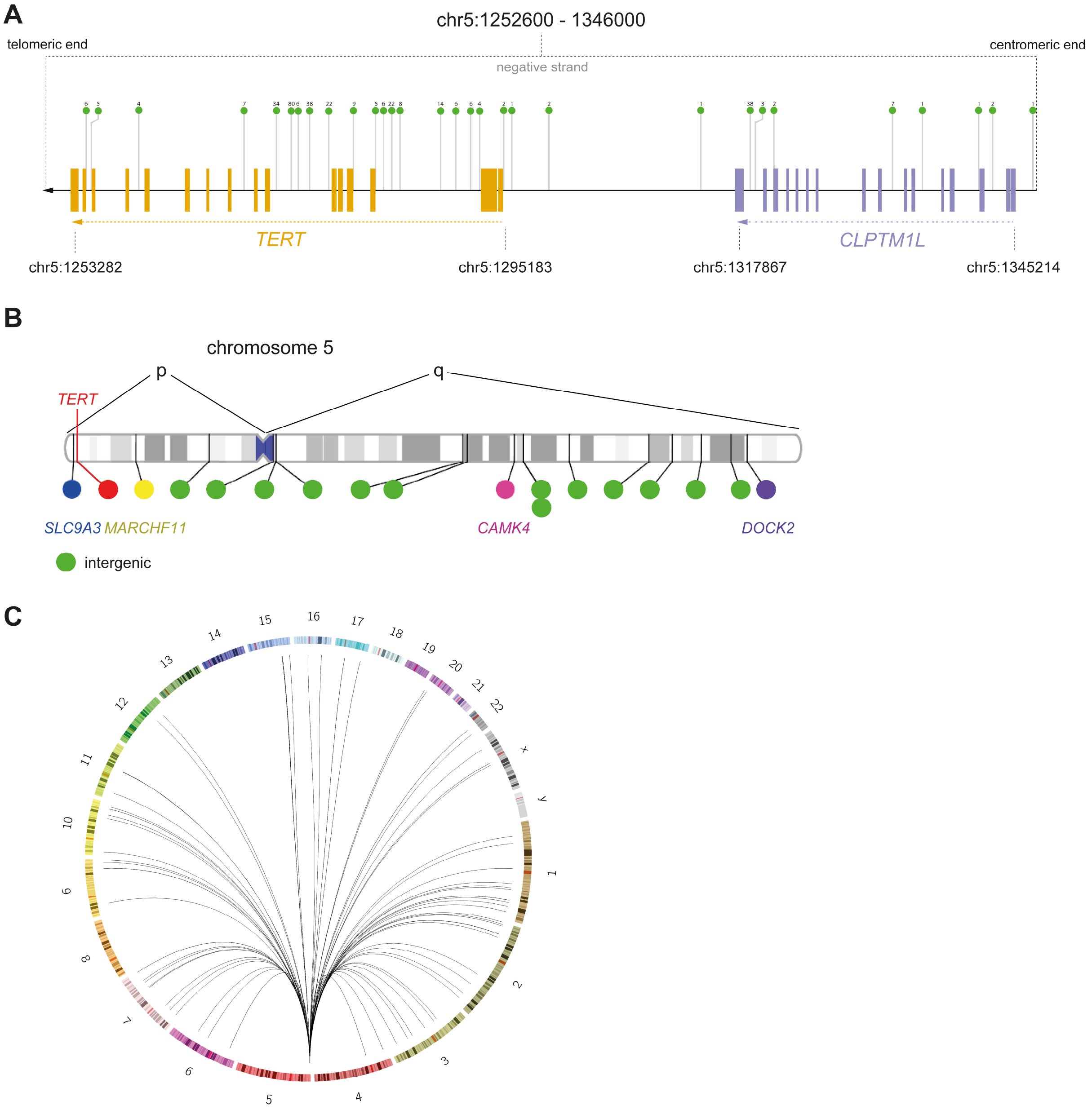

Fig. S1. The rearranged *TERT* region is translocated to a different region in the genome, creating patient-unique genomic breakpoints that are harbored by all or most neuroblastoma cells regardless of their locations. A, Lollipop plot shows distribution of breakpoints involved in rearrangements in the genomic neighborhood of the *TERT* gene (genome assembly GRCh37/hg19: chr5:1252600-1345000, minus strand). Green dots indicate breakpoint locations. Numbers above green dots indicate the number of each breakpoint that was detected. **B**, Ideogram of chromosome 5 displays intrachromosomal *TERT* rearrangement mates. Each dot represents a detected breakpoint to a rearrangement (green: intergenic breakpoint location, colors: breakpoint location is within the correspondingly colored gene on chr5). **C**, Circos plot of interchromosomal translocations from the *TERT* locus to other chromosomes. Lines each depict one translocated fragment from the *TERT* locus.

Supplementary Figure S2

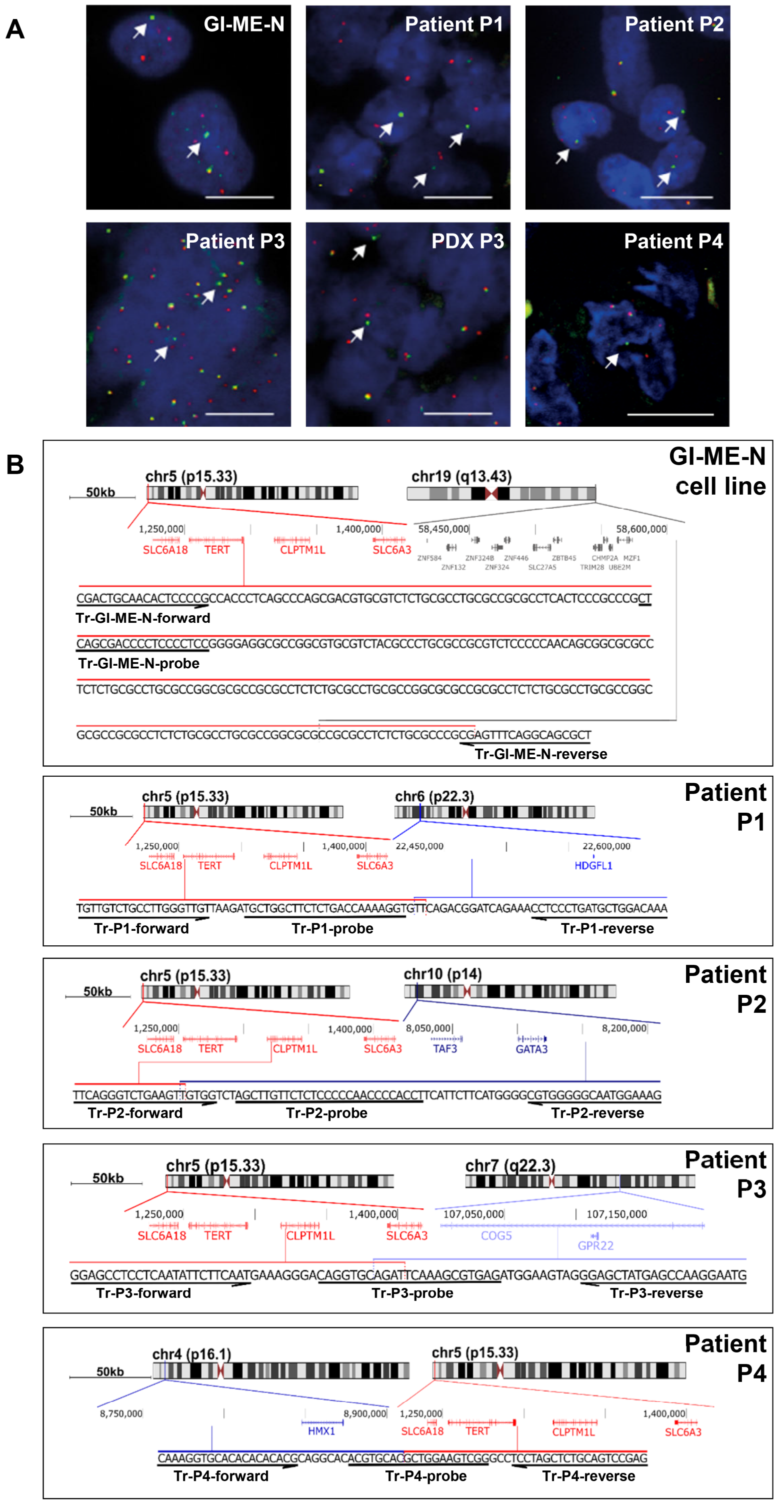

Fig. S2. Characterization of the *TERT* locus in neuroblastoma tissue samples and preclinical disease models. A, *TERT* break-apart FISH results shown for GI-ME-N cells, the diagnostic tumor samples from the indicated patients and a patient-derived xenograft maintained in mice that was established from residual primary tumor tissue collected from patient P3 at diagnosis. A yellow signal indicates that the *TERT* gene (green) and the *CLPTM1L* gene (red) remain in their correct localization next to each other, while a green signal indicates a *TERT* rearrangement, since it is no longer attached to the red-labeled *CLPTM1L* gene. Scale bar=10 µM. **B**, Genome sequences at the *TERT* rearrangement breakpoints (targeted sequencing) are shown with the primer and probe binding sites established for ddPCR-based *TERT* breakpoint detection in GI-ME-N cells and patient tumor tissue samples. The sequence of the *TERT* locus is indicated in red, the rearrangement partner is indicated in grey (GI-ME-N) or blue (tumors).

Supplementary Figure S3

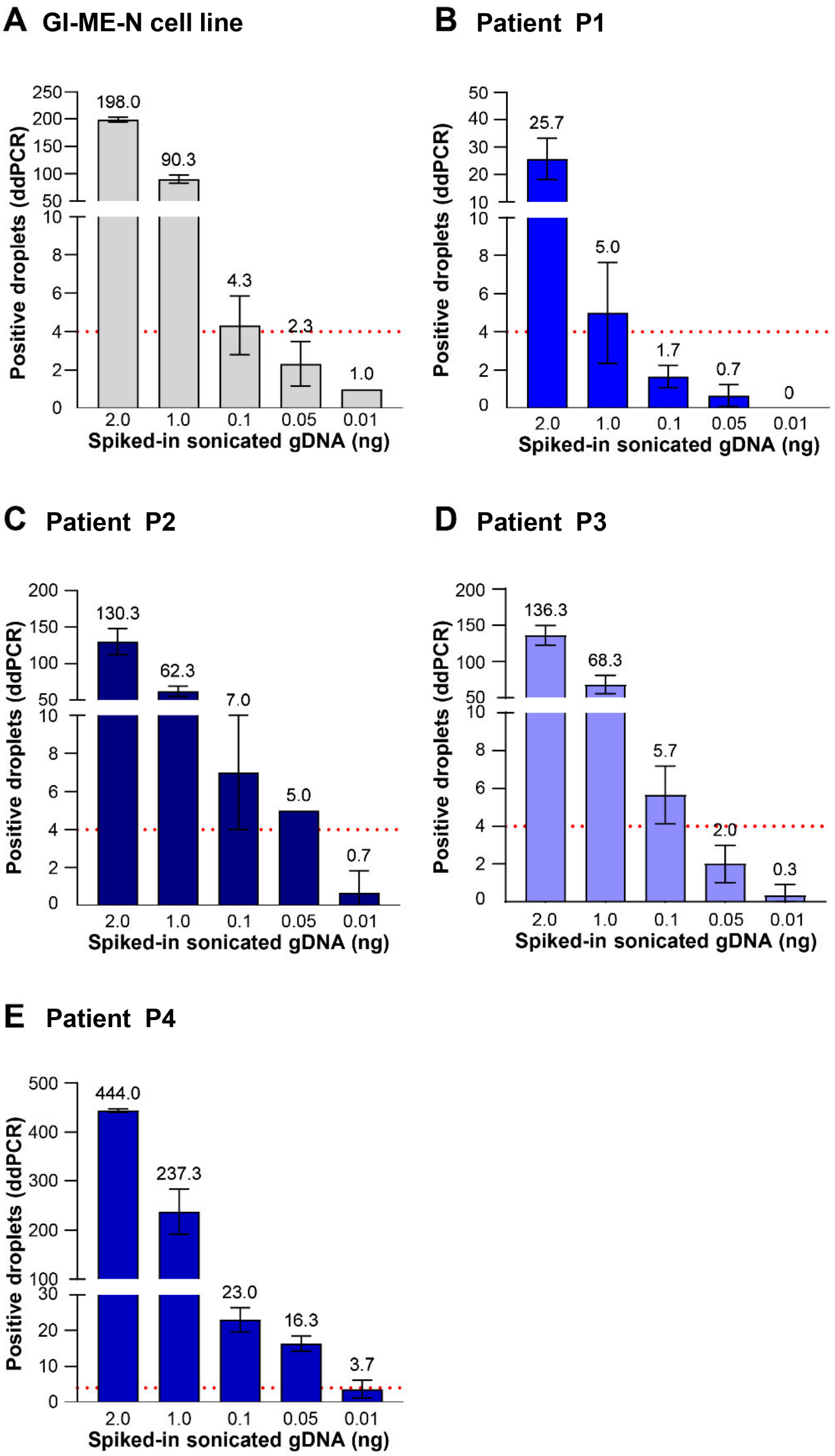

Fig. S3. Limits of ddPCR-based *TERT* breakpoint detection for each unique assay created. Sonicated genomic DNA from GI-ME-N cells (**A**) and restriction enzyme-digested DNA from tumor tissue samples (**B**-**E**) was spiked into pooled blood plasma from 11 pediatric patients with non-malignant conditions in the range of 0.01 – 2.0 ng, and bar graphs show the number of droplets in which the PCR assay was positive (mean ±SD, n ≥ 3). A sample was scored as positive for *TERT* breakpoint detection if the number of positive droplets was ≥ 4 (*red dashed line*). Scale for droplet number is discontinuous to allow full data presentation.

Supplementary Figure S4

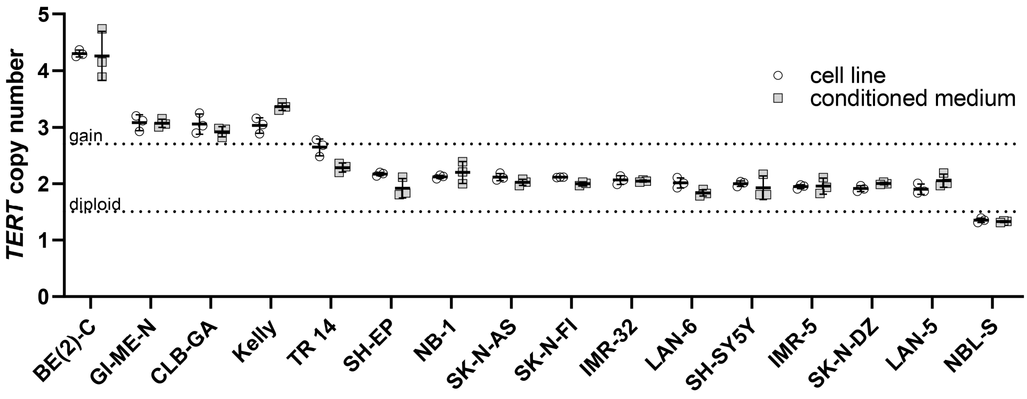

Fig. S4. *TERT* copy numbers in human neuroblastoma cell lines determined from genomic and cell-free DNA by triplex ddPCR. Genomic DNA was extracted from cultured cells and fragmented by sonication before ddPCR for *TERT* copy number (white circles, mean indicated by the line and SD indicated by the whiskers; $n \geq 3$). Cell-free DNA was purified from medium conditioned by the cell lines, and *TERT* copy number was determined by ddPCR (green squares, mean indicated by the line and SD indicated by the whiskers; $n \geq 1$). Dashed lines indicate the copy number thresholds: diploid *TERT* (1.50-2.73 copies), *TERT* gain (2.74 to 8.00 copies).
