# Supplementary material for: Serially Quantifying TERT Rearrangement Breakpoints in ctDNA Enables Minimal Residual Disease Monitoring in Patients with Neuroblastoma
Source: Cancer Res Commun. 2025 Jan 28;5(1):167–77. doi: 10.1158/2767-9764.CRC-24-0569 (PMC11774142; doi:10.1158/2767-9764.CRC-24-0569)
Supplement: Figure S1 [file crc-24-0569_figure_s1_suppsf1.pdf]

FIGURES

Supplementary Figure S1

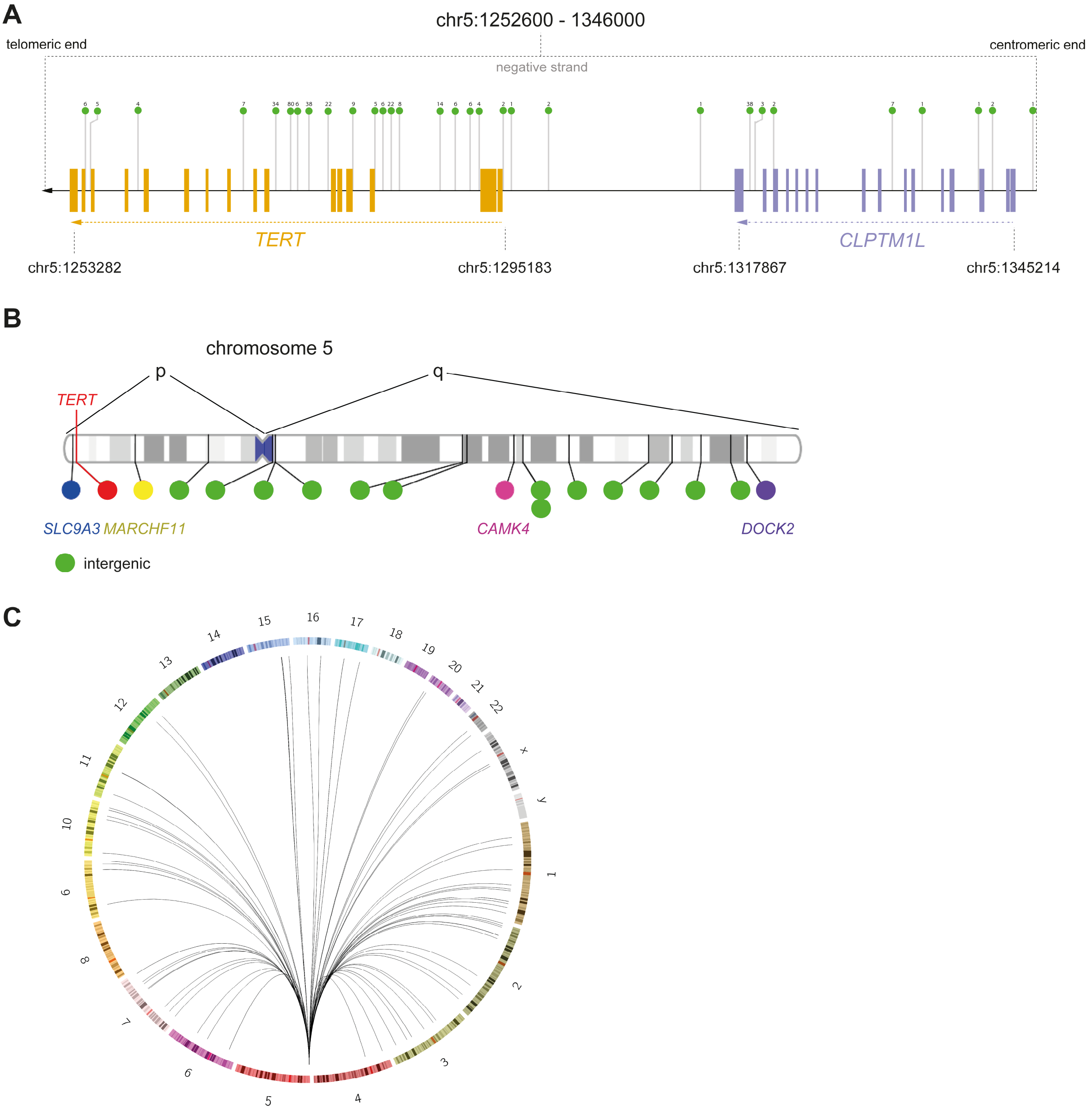

Fig. S1. The rearranged *TERT* region is translocated to a different region in the genome, creating patient-unique genomic breakpoints that are harbored by all or most neuroblastoma cells regardless of their locations. A, Lollipop plot shows distribution of breakpoints involved in rearrangements in the genomic neighborhood of the *TERT* gene (genome assembly GRCh37/hg19: chr5:1252600-1345000, minus strand). Green dots indicate breakpoint locations. Numbers above green dots indicate the number of each breakpoint that was detected. **B**, Ideogram of chromosome 5 displays intrachromosomal *TERT* rearrangement mates. Each dot represents a detected breakpoint to a rearrangement (green: intergenic breakpoint location, colors: breakpoint location is within the correspondingly colored gene on chr5). **C**, Circos plot of interchromosomal translocations from the *TERT* locus to other chromosomes. Lines each depict one translocated fragment from the *TERT* locus.
