# Supplementary material for: Serially Quantifying TERT Rearrangement Breakpoints in ctDNA Enables Minimal Residual Disease Monitoring in Patients with Neuroblastoma
Source: Cancer Res Commun. 2025 Jan 28;5(1):167–77. doi: 10.1158/2767-9764.CRC-24-0569 (PMC11774142; doi:10.1158/2767-9764.CRC-24-0569)
Supplement: Figure S2 [file crc-24-0569_figure_s2_suppsf2.pdf]

Supplementary Figure S2

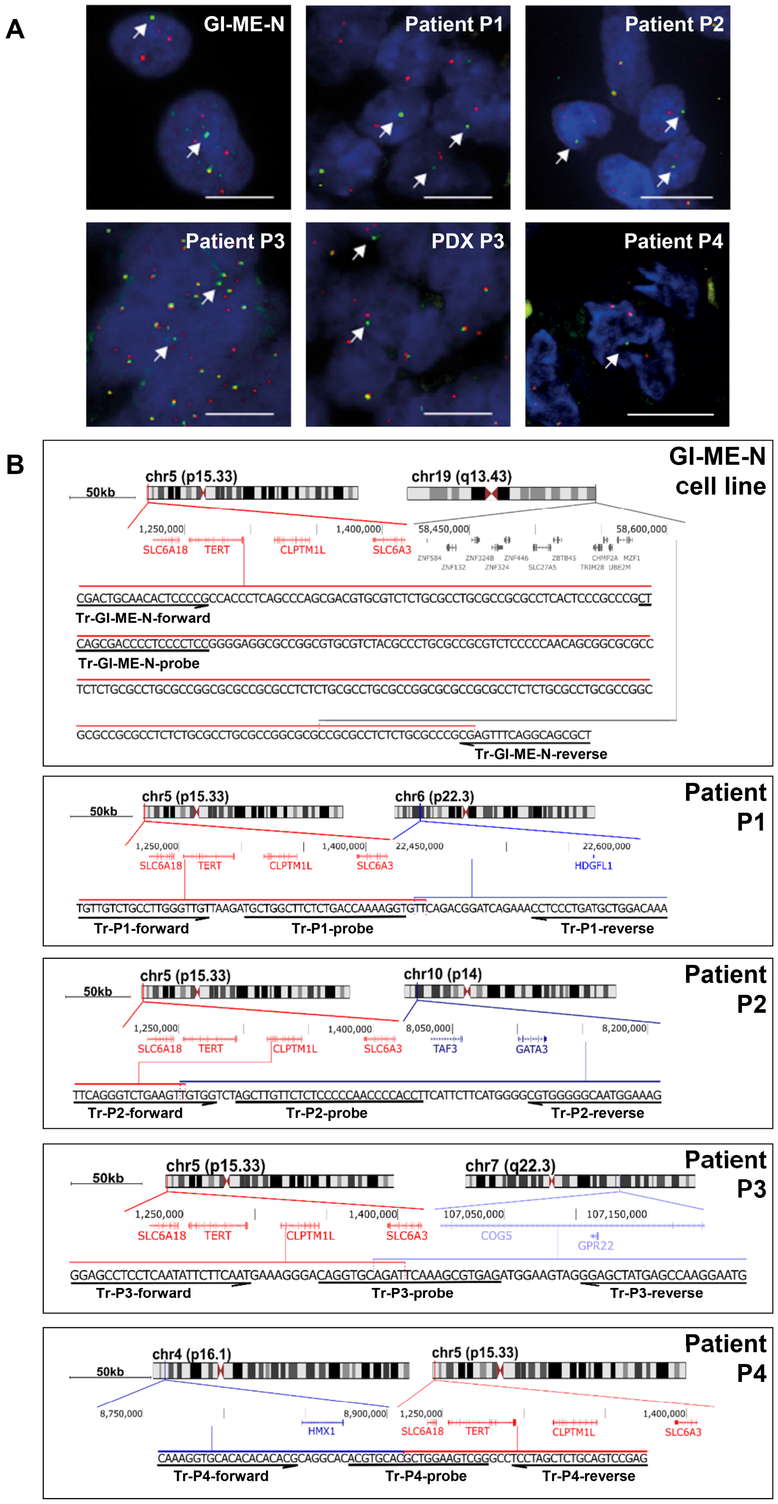

Fig. S2. Characterization of the *TERT* locus in neuroblastoma tissue samples and preclinical disease models. A, *TERT* break-apart FISH results shown for GI-ME-N cells, the diagnostic tumor samples from the indicated patients and a patient-derived xenograft maintained in mice that was established from residual primary tumor tissue collected from patient P3 at diagnosis. A yellow signal indicates that the *TERT* gene (green) and the *CLPTM1L* gene (red) remain in their correct localization next to each other, while a green signal indicates a *TERT* rearrangement, since it is no longer attached to the red-labeled *CLPTM1L* gene. Scale bar=10 µM. **B**, Genome sequences at the *TERT* rearrangement breakpoints (targeted sequencing) are shown with the primer and probe binding sites established for ddPCR-based *TERT* breakpoint detection in GI-ME-N cells and patient tumor tissue samples. The sequence of the *TERT* locus is indicated in red, the rearrangement partner is indicated in grey (GI-ME-N) or blue (tumors).
