# Supplementary material for: Serially Quantifying TERT Rearrangement Breakpoints in ctDNA Enables Minimal Residual Disease Monitoring in Patients with Neuroblastoma
Source: Cancer Res Commun. 2025 Jan 28;5(1):167–77. doi: 10.1158/2767-9764.CRC-24-0569 (PMC11774142; doi:10.1158/2767-9764.CRC-24-0569)
Supplement: Figure S3 [file crc-24-0569_figure_s3_suppsf3.pdf]

Supplementary Figure S3

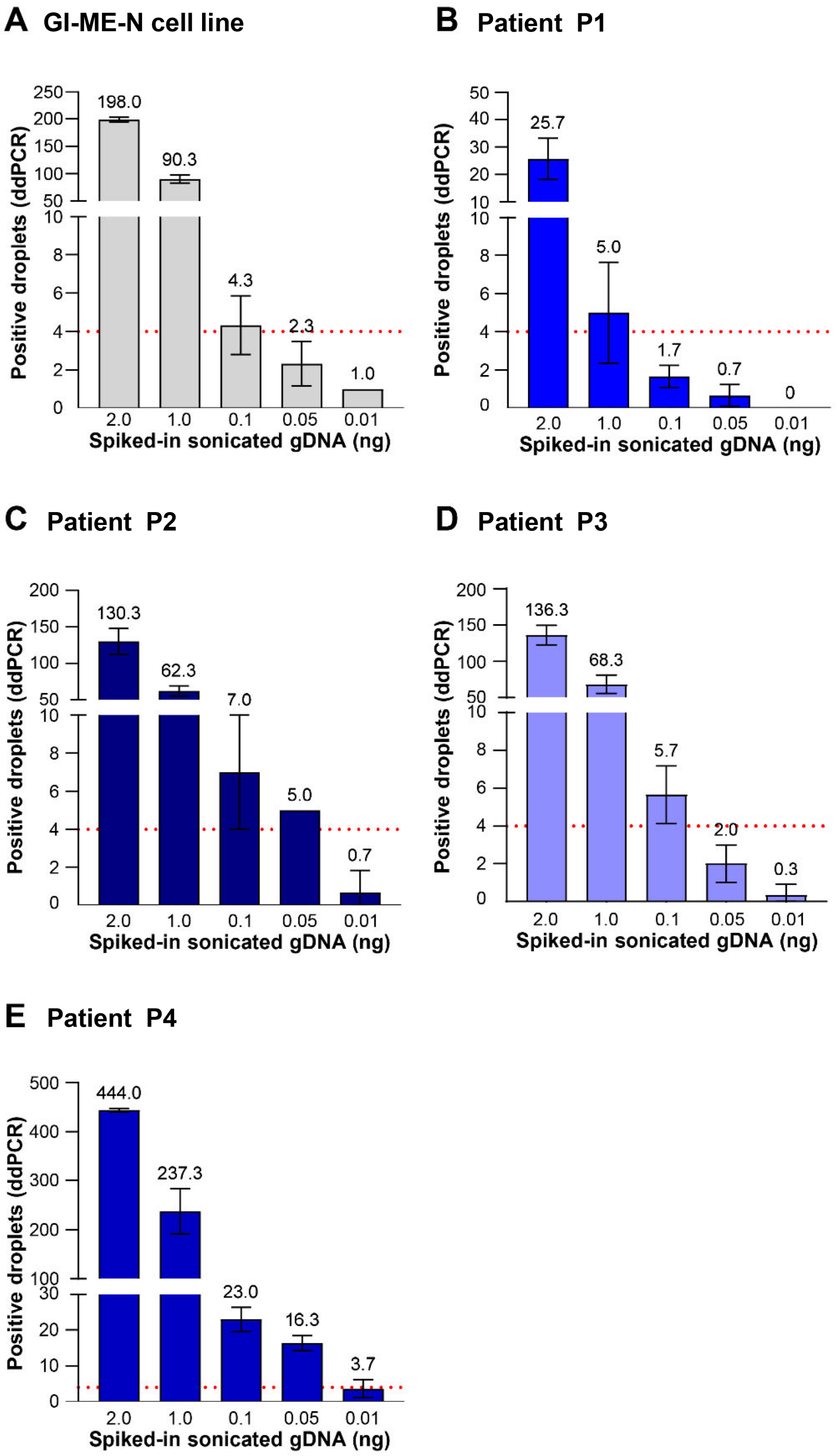

Fig. S3. **Limits of ddPCR-based *TERT* breakpoint detection for each unique assay created**. Sonicated genomic DNA from GI-ME-N cells (**A**) and restriction enzyme-digested DNA from tumor tissue samples (**B**-**E**) was spiked into pooled blood plasma from 11 pediatric patients with non-malignant conditions in the range of 0.01 – 2.0 ng, and bar graphs show the number of droplets in which the PCR assay was positive (mean ±SD, n ≥ 3). A sample was scored as positive for *TERT* breakpoint detection if the number of positive droplets was ≥ 4 (*red dashed line*). Scale for droplet number is discontinuous to allow full data presentation.
