# Supplementary material for: Serially Quantifying TERT Rearrangement Breakpoints in ctDNA Enables Minimal Residual Disease Monitoring in Patients with Neuroblastoma
Source: Cancer Res Commun. 2025 Jan 28;5(1):167–77. doi: 10.1158/2767-9764.CRC-24-0569 (PMC11774142; doi:10.1158/2767-9764.CRC-24-0569)
Supplement: Figure S4 [file crc-24-0569_figure_s4_suppsf4.pdf]

Supplementary Figure S4

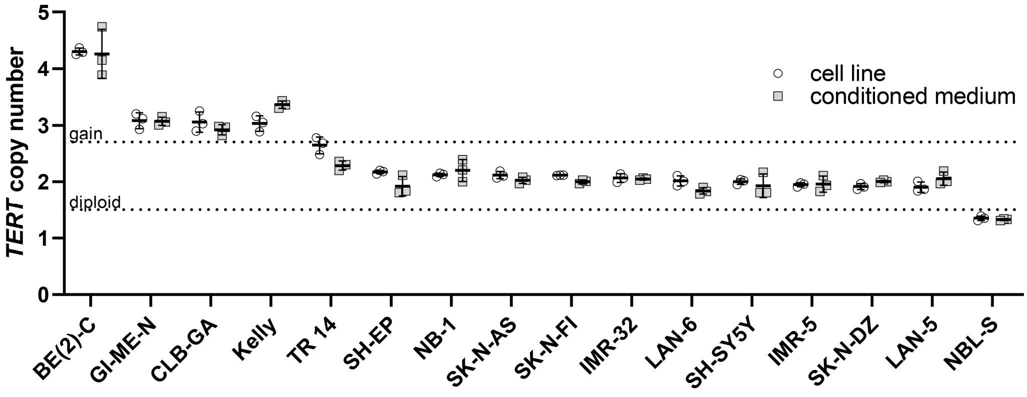

Fig. S4. *TERT* copy numbers in human neuroblastoma cell lines determined from genomic and cell-free DNA by triplex ddPCR. Genomic DNA was extracted from cultured cells and fragmented by sonication before ddPCR for *TERT* copy number (white circles, mean indicated by the line and SD indicated by the whiskers; n ≥ 3). Cell-free DNA was purified from medium conditioned by the cell lines, and *TERT* copy number was determined by ddPCR (green squares, mean indicated by the line and SD indicated by the whiskers; n ≥ 1). Dashed lines indicate the copy number thresholds: diploid *TERT* (1.50-2.73 copies), *TERT* gain (2.74 to 8.00 copies).
